# Oral Hygiene Practices of Hungarian Adult E-Cigarette-Only and Dual Users

**DOI:** 10.3290/j.ohpd.a45520

**Published:** 2020-11-20

**Authors:** Réka Kaán, Melinda Pénzes, Lilla Abafalvi, Péter Hermann, Barbara Kispélyi

**Affiliations:** a Assistant Lecturer, Department of Prosthodontics, Faculty of Dentistry, Semmelweis University, Budapest, Hungary. Idea conception; designed the study, refined the methodology and contributed to the grant application; managed the data collection; drafted and edited the manuscript; read and approved the final manuscript.; b Assistant Professor, Institute of Public Health, Faculty of Medicine, Semmelweis University; Budapest, Hungary. Contributed to the analysis and interpretation of data; drafted and edited the manuscript; read and approved the final manuscript.; c Assistant Lecturer, Department of Prosthodontics, Faculty of Dentistry, Semmelweis University, Budapest, Hungary. Idea conception; designed the study, refined the methodology and contributed to the grant application; managed the data collection; drafted the manuscript; read and approved the final manuscript.; d Professor, Head of Department, Department of Prosthodontics, Faculty of Dentistry, Semmelweis University, Budapest, Hungary. Principal researcher for the project; idea conception; designed the study, refined the methodology and contributed to the grant application; critical revision of the manuscript; read and approved the final manuscript.; e Associate Professor, Department of Prosthodontics, Faculty of Dentistry, Semmelweis University, Budapest, Hungary. Idea conception; designed the study, refined the methodology and contributed to the grant application; critical revision of the manuscript; read and approved the final manuscript.

**Keywords:** e-cigarettes, oral hygiene, tobacco smoking, vaping

## Abstract

**Purpose:**

This study aimed to explore self-reported oral hygiene practices (OHPs) among Hungarian adult e-cigarette-only (former smokers who switched completely to e-cigarette use or vaping) and dual users (smokers who use e-cigarettes and combustible tobacco cigarettes concomitantly).

**Materials and Methods:**

A cross-sectional, web-based survey of 930 adult Hungarian e-cigarette users was conducted in 2015. Participants reported 10 OHPs, which were included in analyses as separate binary variables and as a composite variable of the 10 OHP items (inadequate/adequate). Chi-square test was used to explore whether separate OHPs differ by vaping status, and to examine the relationship between inadequate OHPs and past combustible or e-cigarette use characteristics. Associations between separate OHPs and vaping status, and between inadequate OHPs and vaping status were tested by multiple logistic regression analyses.

**Results:**

More dual users reported toothbrushing twice a day or more than e-cigarette-only users (73.6% vs 65.3%, respectively, p = 0.041) and using sugar-free chewing gum (57.7% vs 45.8%, respectively, p = 0.006) while adequacy of other OHPs did not differ statistically significantly by vaping status. Inadequate OHPs were more typical in the sample (63.7%) than adequate OHPs, however, inadequate OHPs did not differ statistically significantly among dual users and e-cigarette-only users (62.0% vs 64.0%, respectively, OR = 1.20, p = 0.400), controlling for age, gender, education, past combustible and current e-cigarette use characteristics.

**Conclusion:**

In this study, both e-cigarette-only and dual users demonstrated similarly high prevalence of inadequate OHPs. Therefore dentists should educate them about effective OHPs and the role of tobacco and e-cigarette use in the development of oral diseases.

Electronic cigarettes (e-cigarettes) are a subcategory of electronic nicotine delivery systems (ENDS) with a wide range of product design and hundreds of different brands on the market.^9^,^31^ E-cigarettes are battery-powered devices with a heating element (atomiser) that vaporise a liquid solution (e-liquid) containing varying amounts of nicotine, flavourings, humectants (eg, propylene glycol, glycerine) into an aerosol (vapour) which is inhaled through a mouthpiece by the user (vaping).^14^,^31^ The most commonly reported reasons for vaping by adult users include to aid in smoking cessation or harm reduction, to cope with tobacco craving, to avoid smoking restrictions, and to protect non-smokers from second-hand smoke exposure.^9^ Despite the indisputably increasing popularity of e-cigarettes, there are ongoing debates in the public health community on the potential harms or benefits of vaping. Advocates of harm reduction emphasise that e-cigarettes have the potential to encourage combustible tobacco smoking reduction (aka dual use of combustible cigarettes and e-cigarettes) or cessation.^4^,^31^ In contrast, opponents of harm reduction argue that e-cigarettes may be promoting harm escalation by greater nicotine intake through dual use and consequently prolonging tobacco use. Furthermore, the long-term health risks of vaping are currently unknown.^4^,^31^

There are scarce data about e-cigarette use in Hungary. According to a recent Eurobarometer survey, both ever use and current use of e-cigarettes were lower in Hungary (10% and 1%, respectively) compared to the European Union average (16% and 2%, respectively) among 15-year-old and older respondents.^5^ Regarding Hungarian current smokers and ex-smokers in this survey, ever use of e-cigarettes were 19% and 13%, respectively, while current use was indicated to be 0% and 3%, respectively.^5^

Adult e-cigarette users commonly perceive e-cigarettes as a less harmful and a safer alternative to combustible cigarettes,^9^ however, there are many uncertainties regarding the absolute harm and both short-term and long-term health effects of vaping.^4^ Besides that, a growing body of literature documents the effect of vaping on the pulmonary and cardiovascular systems as well as the cytotoxicity of e-cigarette aerosol,^9^ recent studies also indicate that e-cigarette use is not without consequences for oral health. A population-based cross-sectional study among US adults found that daily e-cigarette use was associated with poor oral health outcomes including periodontal diseases and tooth loss.^11^ Kim et al^15^ assessed the cariogenic potential of e-cigarette aerosols generated from sweet flavoured e-liquids in an in vitro study. They detected that viscous e-cigarette aerosol covers enamel surface similar to high-sucrose candies and acidic drinks which promotes *Streptococcus mutans* attachment, especially in vapour-exposed pits and fissures. Furthermore, specific flavours and chemical by-products of e-liquids provide an additional food source to pathogenic oral bacteria, contributing to biofilm formation and consequently demineralisation of the enamel.^15^ Moreover, varying amounts of multiple sugars, including sucrose, fructose and glucose, were detected in unheated, fruit and dessert-flavoured e-liquids,^6^ although the sugar content of the inhaled vapour and its cariogenic potential is currently unknown. It is assumed that vaping also impacts on dental aesthetics, that is, some e-liquid flavours cause perceptible tooth discoloration.^19^ Few in vitro studies assessed the effect of vaping on the periodontium and explored several possible pathways of how e-cigarette use may contribute to periodontitis. Nicotine and various flavouring chemicals in the vapour may damage periodontal ligament fibroblasts, gingival fibroblasts and oral myofibroblasts due to oxidative stress-induced cellular senescence, DNA damage, dysregulated repair mechanism, and altered inflammation leading to periodontal diseases.^12^ Furthermore, inhaled nicotine from the vapour may promote biofilm formation, later pathogen bacteria attachment on tooth surfaces, accumulation of tooth calculus and the development of caries and periodontal diseases in e-cigarette users similarly to tobacco cigarette smokers.^10^,^17^

Appropriate oral hygiene practices (OHPs) are crucial for dental plaque control to prevent caries and periodontal diseases.^21^ General oral hygiene recommendations for adult persons include toothbrushing with fluoridated toothpaste and either a manual or powered toothbrush at least twice daily, interdental plaque control daily with dental floss or an interdental brush, using fluoride mouthrinse daily at a different time to brushing, reducing the frequency of sugary food and drinks, and visiting the dentist regularly.^21^,^22^ Additionally, regular use of sugar-free chewing gum can also be beneficial in maintaining oral health.^3^

Tobacco smokers have an increased risk for periodontal diseases and tooth loss which is possibly due to their lower compliance with OHPs, besides the negative effects of cigarette smoke compounds on oral tissues.^10^,^13^,^16^,^17^,^23^,^25^ Existing studies indicate that e-cigarette use may also have an unfavourable impact on oral health, however, it is currently unknown whether OHPs of e-cigarette users differs from combustible cigarette smokers. Differences in OHPs between e-cigarette users and tobacco smokers can influence the incidence of oral diseases in the long term, hence, dental professionals should be aware of tobacco smoker and/or e-cigarette user patients’ OHPs in order to motivate them for improving their oral hygiene. Common reasons for e-cigarette use are tobacco harm reduction and the perception that e-cigarettes are less toxic than tobacco smoking.^9^ After switching completely from tobacco cigarettes to e-cigarettes, many e-cigarette users report improvements in their specific physiological functions and in overall health.^1^ We hypothesised that e-cigarette-only users, who supposed to be more health conscious and committed to improve both their overall and oral health, have better oral health practices than dual users. Therefore, this study aims to explore self-reported OHPs among Hungarian adult e-cigarette-only users (former smokers who switched completely to e-cigarettes) and dual users (smokers who use e-cigarettes and combustible tobacco cigarettes concomitantly), and to assess the relationship between inadequate OHPs, past conventional and current e-cigarette use characteristics.

## Materials and Methods

### Participants and Procedure

We conducted a cross-sectional online survey among adult (age 18+) Hungarian vapers in September–December, 2015. The convenience sample was obtained by posting the survey on Hungarian e-cigarette forum websites and an e-cigarette webshop inviting website visitors to participate.^1^ After reading the description of the study, participants consented to participate by voluntarily answering the survey questions. In the first 2 months of the study, 800 participants completed the survey anonymously, then after 2 months, a lottery-based incentive was offered to increase participation and further 784 participants completed the survey, indicating their e-mail address to participate in the lottery. The study was approved by the Institutional Review Board of Semmelweis University, Budapest.

Of the 1,584 initial respondents, we excluded those who were <18-year-old (n = 4), had never smoked (n = 22), did not respond whether they use only e-cigarette and/or combustible cigarette (n = 63), responded inconsistently to questions assessing e-cigarette-only and dual use (n = 40), and did not respond to all oral hygiene-related questions (n = 112). Since we did not have access to respondents’ internet protocol (IP) address to exclude multiple responses from the same participants, we searched for duplicate cases (n = 413) using all sociodemographic variables (gender, age, education level, type of settlement, and income level). We applied an *a priori* decision rule that only the first case of potential duplicate respondents was included in the final analytical sample. As a result, 930 unique respondents who ever smoked and were current e-cigarette users (only or dual) were included in this study.

### Measures

The electronic self-administered questionnaire consisted of seven parts, including questions about respondents’ sociodemographic characteristics, e-cigarette use, combustible cigarette use, perceived harm of e-cigarettes, oral hygiene habits, vaping-related adverse events, and changes in physiological functions since initiating e-cigarette use.^1^ For the current study, the following variables were included.

Sociodemographic data were collected on gender, age (range 18–75, mean age 38.2 [SD = 11.5], collapsed into 18–34, 35–49, and 50+ year-old categories), and education (technical school or less – without graduation certificate, high school or vocational school – with graduation certificate, and college or university).

E-cigarette-only versus dual use was assessed by the question: ‘Do you use an e-cigarette or combustible cigarette?’ (combustible cigarettes only, e-cigarettes only, both of them). Only persons who were e-cigarette-only users and dual users were included in the study.

Past combustible cigarette use was measured by the number of tobacco cigarettes smoked per day before initiating e-cigarette use. Response options were categorised into: ≤10 cigarettes per day (CPD) – light smoker; 11–19 CPD – moderate smoker; and ≥20 CPD – heavy smoker.

Current e-cigarette use characteristics variables included in this study were (1) time since respondent started using e-cigarettes (<6 month ago, 6–12 months ago, 1–2 years ago, >2 years ago); (2) frequency of e-cigarette use per day (non-daily, 1–10 times a day, 11–19 times a day, ≥20 times a day); (3) nicotine concentration of the e-liquid (0 mg – nicotine-free, 1–6 mg – low, 7–12 mg – medium, ≥13 mg – high; and (4) e-liquid flavour category (tobacco, fruit and/or dessert) (Table 1).

**Table 1:** Descriptive characteristics of the sample by e-cigarette use status

Variable	Total n = 930	E-cigarette-only user, n (%) 767 (82.5)	Dual user, n (%) 163 (17.5)	Statistic (p value)
**Gender**				
Male	769 (83.1)	635 (83.3)	134 (82.2)	0.728
Female	156 (16.9)	127 (16.7)	29 (17.8)	
**Age (years)**				
18–34	382 (41.1)	313 (40.8)	69 (42.3)	0.228
35–49	392 (42.2)	318 (41.5)	74 (45.4)	
50+	156 (16.8)	136 (17.7)	20 (12.3)	
**Education**				
Technical school or less (without graduation certificate)	213 (24.9)	178 (25.3)	35 (23.0)	0.174
Gymnasium or vocational school (with graduation certificate)	371 (43.3)	312 (44.3)	59 (38.8)	
University or college	272 (31.8)	214 (30.4)	58 (38.2)	
**Time since started using e-cigarette**				
Less than 6 months ago	172 (18.7)	128 (16.8)	44 (27.5)	0.004
6–12 months	171 (18.6)	142 (18.7)	29 (18.1)	
1–2 years	200 (21.7)	162 (21.3)	38 (23.8)	
More than 2 years ago	377 (41.0)	328 (43.2)	49 (30.6)	
**Frequency of e-cigarette use**				
Non-daily	20 (2.2)	6 (0.8)	14 (8.7)	<0.001
1–10 times a day	81 (8.9)	56 (7.4)	25 (15.5)	
11–19 times a day	214 (23.4)	169 (22.4)	45 (28.0)	
≥20 times a day	599 (65.5)	522 (69.3)	77 (47.8)	
**Tobacco cigarettes smoked per day (before starting use of e-cigarette)**				
≤10 cigarettes a day	75 (8.1)	56 (7.3)	19 (11.9)	0.004
11–19 cigarettes a day	240 (25.9)	187 (24.4)	53 (33.3)	
≥20 cigarettes a day	611 (66.0)	524 (68.3)	87 (54.7)	
**Nicotine concentration of the e-liquid**				
0 mg	60 (6.5)	46 (6.1)	14 (8.6)	0.023
1–6 mg	461 (50.1)	397 (52.3)	64 (39.5)	
7–12 mg	317 (34.4)	248 (32.7)	69 (42.6)	
≥ 13 mg	83 (9.0)	68 (9.0)	15 (9.3)	
**E-liquid flavour category**				
Tobacco	157 (17.7)	119 (16.2)	38 (24.8)	0.011
Fruit and/or dessert	731 (82.3)	616 (83.8)	115 (75.2)	

Participants self-reported 10 OHPs (see Table 2) which were included in analyses as separate binary variables, as well as a composite variable that was created by combining the 10 binary OHP items. The added score of the composite variable ranged from 1 to 8 and was further collapsed into a binary adequacy of OHPs variable by using median split method (scores 1–4 = inadequate OHPs; scores 5–8 = adequate OHPs).^13^

**Table 2:** Associations between oral hygiene practices and vaping status in multiple binary logistic regression models

Oral hygiene practices (OHPs) dependent variables	Total (%)	EC-only users (%)	Dual users (%)	p value^a^	OR [CI95%]^b^
Toothbrushing (≥2x/day)	66.8	65.3	73.6	0.041	0.66 [0.45–0.97]
Toothpaste (fluoride)	62.6	63.9	56.4	0.075	1.36 [0.96–1.91]
Chewing gum (yes, sugar-free)	47.8	45.8	57.7	0.006	0.64 [0.45–0.90]
Consume sweets/sugary drinks (<3–4×/week)	69.8	69.9	69.3	0.888	1.00 [0.69–1.44]
Type of toothbrush (electronic/electronic and conventional)	24.3	23.7	27.0	0.377	0.83 [0.57–1.22]
Use of oral care device other than toothbrush and toothpaste (yes)	41.7	40.9	45.4	0.294	0.82 [0.59–1.16]
Use of dental floss/interdental brush (yes)	22.4	22.0	23.9	0.598	1.00 [0.60–1.34]
Use of mouthwash (yes)	47.0	48.2	41.1	0.097	1.35 [0.96–1.90]
Use of other oral care device (yes)	4.1	4.3	3.1	0.469	1.43 [0.55–3.75]
Dental visits (twice a year)	17.5	17.7	16.6	0.722	1.10 [0.70–1.73]

EC-only, e-cigarette-only; ^a^p value of χ^2^ test; ^b^ Multiple binary logistic regression models; all models were controlled for gender and age; reference: dual users.

### Statistical Analysis

Pearson’s chi-square test was used to describe the characteristics of the sample, to explore whether separate OHPs differ by vaping status, and to examine the relationship between inadequate OHPs and past combustible or e-cigarette use characteristics. Multiple logistic regression analyses were used to test associations between separate OHPs and vaping status, adjusting all models for age (as continuous variable) and gender. The association between adequacy of OHPs and vaping status was also tested by multiple logistic regression analysis, controlling for age, gender, education, past conventional and current e-cigarette use characteristics. All analyses were performed using IBM SPSS version 24.0 software, and statistical significance level was accepted at p <0.05.

## Results

Descriptive characteristics of the sample by vaping status are presented in Table 1. Compared to dual users (17.5%), e-cigarette-only users (82.5%) were more likely initiating e-cigarette use more than 2 years prior to the survey (30.6% vs 43.2%, respectively, p = 0.004). More than two-thirds of e-cigarette-only users reported vaping ≥20 times a day, while only half of dual users responded similarly (p <0.001). E-cigarette-only users preferred low nicotine concentration e-liquid (52.3%) and fruit and/or dessert flavoured e-liquids (83.8%) more likely than dual users (39.5%, p = 0.023, and 75.2%, p = 0.011, respectively). Past heavy smoking before initiating vaping was more common among e-cigarette-only users than dual users (68.3% vs 54.7%, respectively, p = 0.004). Sociodemographic characteristics did not differ statistically significantly by vaping status, although men were overrepresented while the 50+ age group was underrepresented in the sample, and approximately one-third of respondents had a college/university degree.

Table 2 describes the adequacy of separate OHPs by vaping status, and presents the associations between OHPs and vaping status based on multiple logistic regression models. More dual users reported toothbrushing twice a day or more than e-cigarette-only users (73.6% vs 65.3%, respectively; OR = 0.66, CI95%: 0.45–0.97, p = 0.035) and using sugar-free chewing gum (57.7% vs 45.8%, respectively; OR = 0.64, CI95%: 0.45–0.90, p = 0.010) while adequacy of other OHPs did not differ statistically significantly by vaping status. Approximately two-thirds of the sample reported adequate compliance with some OHPs, that is, consuming sweets/sugary drinks <3–4 times/week (69.8%), toothbrushing ≥2 times/day (66.8%), and using fluoride toothpaste (62.6%). Almost half of respondents indicated properly consuming sugar-free chewing gum (47.8%) and using mouthwash regularly (47.0%). Only 24.3% of the sample used an electronic/electronic and conventional toothbrush, while using dental floss or an interdental brush were even less common (22.4%). Recommended dental visits twice a year was accomplished by a minority of respondents (17.5%) and using oral care devices other than the listed were rare (4.1%).

In overall, inadequate OHPs were more typical in the sample (63.7%) than adequate OHPs, and statistically significantly more likely among males (p = 0.039), 50+ year-old respondents (p = 0.007) and participants with technical school or less education (p <0.001) (Table 3). Including vaping status into analyses, male sex (p = 0.036) and the 50+ year-old age group (p = 0.023) showed statistically significant association with inadequate OHPs in the e-cigarette-only user group while inadequate OHPs were statistically significantly more common both among less educated e-cigarette-only (p <0.001) and dual users (p = 0.016). Inadequate OHPs were more common among past heavy smokers than light and moderate smokers, but only among dual users (p = 0.049). Duration and frequency of e-cigarette use, nicotine concentration of the e-liquid and preferred e-liquid flavour category did not show a statistically significant impact on OHPs. In multiple logistic regression analysis, inadequate OHPs did not differ statistically significantly among dual users and e-cigarette-only users (62.0% vs 64.0%, respectively; OR = 1.20, CI95%: 0.78–1.84, p = 0.400), controlling for age, gender, education, past combustible cigarette and current e-cigarette use characteristics.

**Table 3:** Inadequate oral hygiene practices by sociodemographic, combustible cigarette and e-cigarette use characteristics among e-cigarette users

Variable	Total (%)	EC-only user (%)	Dual user (%)
**Gender**			
Male	65.1*	65.7*	62.7
Female	56.4	55.9	58.6
**Age (years)**			
18–34	66.2*	66.1*	66.7
35–49	58.2	58.8	55.4
50+	71.2	71.3	70.0
**Education**			
Technical school or less (without graduation certificate)	75.6*	75.3*	77.1*
Gymnasium or vocational school (with graduation certificate)	68.2	67.9	69.5
University or college	50.0	50.0	50.0
**Time since started using e-cigarette**			
Less than 6 months ago	66.3	68.0	61.4
6–12 months	68.4	68.3	69.0
1–2 years	58.0	58.6	55.3
More than 2 years ago	63.4	63.4	63.3
**Frequency of e-cigarette use**			
Non-daily	55.0	33.3	64.3
1–10 times a day	58.0	60.7	52.0
11–19 times a day	59.8	61.5	53.3
≥20 times a day	65.8	65.1	70.1
**Tobacco cigarettes smoked per day (before started using e-cigarette)**			
≤10 cigarettes a day	58.7	62.5	47.4*
11–19 cigarettes a day	62.5	65.2	52.8
≥20 cigarettes a day	64.6	63.7	70.1
**Nicotine concentration of the e-liquid**			
0 mg	61.7	63.0	57.1
1–6 mg	62.7	62.2	65.6
7–12 mg	63.1	64.1	59.4
≥ 13 mg	74.7	76.5	66.7
**E-liquid flavour category**			
Tobacco	65.0	65.5	63.2
Fruit and/or dessert	63.5	63.5	63.5

Binary oral hygiene practices (OHPs) variable was used in χ^2^ test analyses (inadequate OHPs/adequate OHPs); EC-only, e-cigarette-only; *p < 0.05.

## Discussion

Our study explored mostly similar patterns of separate OHPs among e-cigarette-only and dual users, and in overall, inadequate OHPs were self-reported by two-thirds of both e-cigarette user groups. The prevalence of separate OHPs were in concordance with a recent representative Hungarian survey, which detected similar frequencies for twice a day toothbrushing (65%), electronic toothbrush use (17%), mouthwash use (42%), and dental floss or interdental brush use (26%) among 14–50-year-old Hungarian respondents.^7^ Some studies found that toothbrushing frequency is poorer among smokers than non-smokers and ex-smokers,^23^,^26^,^28^ however toothbrushing twice daily and using chewing gum were more frequent among dual users compared to e-cigarette-only users in our sample. A possible explanation for these could be that e-cigarette-only users may perceive e-cigarettes safe for oral health and less malodorous than combustible cigarettes, while dual users probably mask their halitosis with regular toothbrushing and chewing gum use.

The strong association between tobacco smoking and periodontitis incidence and progression is well known,^16^ while recent studies indicated that vaping may also contribute to the development of periodontal diseases.^12^ Some studies suggest that both tobacco smoking and e-cigarette use may increase the risk of dental caries.^2^,^15^ Plaque accumulation and subsequent gingivitis can progress to periodontitis, however electronic toothbrushes can reduce plaque and gingivitis more effectively than manual toothbrushing both in the short and long term.^32^ Higher costs of powered toothbrushes and its unrecognised benefits may explain the low electronic/electronic and conventional toothbrush use. Besides toothbrushing, controlling plaque formation by mechanical cleaning of the interproximal tooth surfaces is also important to prevent caries and periodontal diseases.^24^ In our sample, only one-fifth of e-cigarette users indicated using interproximal cleaning aids such as dental floss and interdental brush. Some other studies detected more common regular use of interproximal cleaning aids, although age and smoking status might influence their use.^23^,^24^ Santos et al^23^ found that heavy smokers use dental floss less frequently than other smoking groups, but interdental brush use did not differ by smoking status. Poor interproximal cleaning practice may be associated with the predominantly past heavy smoking habit in our sample. In addition to mechanical cleaning, chemical plaque control with antibacterial mouthwash would be valuable to prevent gingivitis and subsequent periodontal diseases.^20^ However, for smokers, an important drive for mouthwash use could be decreasing halitosis due to tobacco use.^18^ A Scottish study detected that smokers were more likely to use mouthwash regularly (53.1%) compared to never-smokers (40.3%),^18^ although another study conducted among Spanish patients diagnosed with periodontal disease did not show similar association.^23^ Contrary to these findings, in our sample regular mouthwash use was self-reported slightly more often by e-cigarette-only users than dual users, which is possibly due to a more health conscious behaviour of e-cigarette-only users as well as to the better availability, promotion and easier use of mouthwash than other oral care devices. Although frequent consumption of sweets and sugary soft drinks was slightly favourable in our overall sample (30.4%) compared to the relevant Hungarian data of a recent Eurobarometer survey (22–25%), both national findings outweighed the European Union average consumption data (15–19%).^29^ Furthermore, the majority of e-cigarette users preferred fruit and/or dessert flavoured e-liquids in our sample. High amount and frequent consumption of free sugars is a major risk factor for caries development, and in addition, certain sweet and fruity e-liquid flavours with high potential for cariogenicity as well as varying amounts of sugars in flavoured e-liquids may also increase cariogenic potential.^2^,^6^,^15^ Moreover, more than 90% of our respondents preferred nicotinic e-liquid, and inhaled nicotine from the vapour may promote the development of caries through bacterial biofilm formation in similar way like nicotine from tobacco smoke.^10^,^17^ Several studies indicated that dental attendance is poorer among smokers compared to non-smokers.^13^,^26^,^28^ Moreover, increasing smoking frequency is associated with decreasing dental visits.^27^ This association might explain the low rate of dental visits in our mostly past heavy smoker sample, that is, only less than one-fifth of our respondents reported twice a year dental visits. Although recall interval of routine dental examinations could be varying depending on individual risk for oral diseases,^22^ both smokers and presumably e-cigarette users have increased risk for developing oral diseases, which may establish the indication of 6-monthly dental examination.^8^

Our results indicate that overall OHPs were far below an acceptable level in both groups of e-cigarette users. Some studies found that tobacco smokers had much poorer oral hygiene compared to non-smokers.^13^,^23^ Furthermore, oral hygiene may differ by smoking intensity, that is, individuals with greater smoking intensity, particularly heavy smokers perform poorer oral hygiene habits.^13^ This association was also detected in our sample, especially among dual users. In addition to smoking, demographic and socioeconomic factors such as male sex, older age and lower educational level also contribute to poorer OHPs,^7^,^26^,^28^,^30^ however, our results demonstrated similar statistically significant association mainly among e-cigarette-only users. To our knowledge, OHPs of e-cigarette users was not investigated to date. A possible explanation for high prevalence of inadequate OHPs among e-cigarette-only users could be that as many e-cigarette users perceive e-cigarettes less harmful than conventional tobacco products,^9^ e-cigarette-only users may also perceive vaping as a universal tool for improving personal health, however, because of their false sense of security, they may underestimate the need for continuous personal health promotion, including the maintenance of good oral health.

This study has some limitations. First, self-reported data are prone to recall and social desirability bias. Second, individuals with more positive perceptions and experiences of vaping may have been more motivated to participate in the survey leading to respondent bias. Third, the cross-sectional design and convenience sample limit causal inference. Finally, this study similar to others, is based on a convenience sample of users, therefore the generalisation of results is limited, however having a representative sample of e-cigarette users is difficult to define, and rarely applied in e-cigarette research. Furthermore, the pattern of e-cigarette use is continuously changing which may also limit the generalizability of our results.^1^

## Conclusion

In conclusion, oral hygiene habits of Hungarian e-cigarette users were poor, and both e-cigarette-only and dual users demonstrated similarly high prevalence of inadequate OHPs. However, further research is required to determine OHPs among different e-cigarette user groups and longitudinal studies are needed to explore more accurately the effect of e-cigarette use on oral health. There are many scientific evidences on the adverse oral health consequences of tobacco smoking,^2^,^16^ while little is known on the short- and long-term effects of e-cigarette use on the oral cavity, although the inhaled high temperature vapour and its toxic components besides nicotine and sweet and fruity flavourings are probably harmful for teeth and oral soft tissues.^12^,^15^ Therefore, graduate and postgraduate education of dental professionals should include brief smoking cessation training with a specific emphasis on the oral health consequences of e-cigarette use. They should be aware of that both tobacco cigarette and e-cigarette use can increase the risk of oral diseases. Besides oral hygiene habits of the patient, past and current smoking as well as e-cigarette use status should be routinely assessed during dental visits. In case of poor oral hygiene, dental professionals should motivate and educate e-cigarette-only and dual user patients to improve their oral hygiene habits. Regarding oral hygiene recommendations for e-cigarette-only users and especially for dual users, dentists should encourage them to switch to powered toothbrushes and using interproximal cleaning aids in order to prevent and treat periodontal diseases. During consultations with patients, dentists should provide balanced information regarding the possible oral health effects of e-cigarettes including periodontal diseases and caries, uncertainties of its use as a cessation aid, and advocate approved cessation supports including nicotine replacement and pharmacotherapy options. Tobacco and/or e-cigarette users are a high-risk group for developing dental diseases, thus the dental team should educate them about the effective oral hygiene, the role of tobacco and e-cigarette use in the development of oral diseases, and the importance of 6-monthly dental check-ups.
